# Kaufman oculocerebrofacial syndrome: case report of a UBE3B splice site variant and clinical overview of reported patients

**DOI:** 10.1186/s13039-025-00742-3

**Published:** 2025-11-30

**Authors:** Abedulrhman S. Abdelfattah, Mohammad Abu Saleh

**Affiliations:** 1 Department of Pediatrics, Faculty of Medicine, The Hashemite University, Zarqa, Jordan; 2 Faculty of Medicine, The Hashemite University, Zarqa, Jordan

**Keywords:** Kaufman oculocerebrofacial syndrome, *UBE3B* gene, Developmental delay, Dysmorphic features, Whole exome sequencing.

## Abstract

**Background:**

Kaufman oculocerebrofacial syndrome (KOS; OMIM #244450)is a rare autosomal recessive disorder caused by pathogenic biallelic variants in *UBE3B*, characterized by craniofacial dysmorphism, global developmental delay, hypotonia, and multisystem anomalies.

**Case presentation:**

We describe a 12-month-old Jordanian girl born to consanguineous parents, who exhibited microcephaly, hypotonia, feeding difficulties, and failure to thrive. Echocardiography revealed a mild basal septal hypertrophy. Developmental evaluation confirmed moderate global delay. Whole-exome sequencing revealed a homozygous *UBE3B* splice site variant (c.1741 + 2T > C), previously reported as pathogenic in ClinVar and classified as pathogenic according to ACMG/AMP criteria but without a detailed phenotypic description. Family history revealed additional neonatal deaths in a consanguineous context, raising the possibility of an underlying autosomal recessive condition.

**Conclusion:**

This case adds to the limited body of literature on KOS and provides further evidence for the pathogenicity of the c.1741 + 2T > C variant. This case highlights the importance of considering KOS in infants presenting with characteristic craniofacial features such as blepharophimosis, ptosis, preauricular tags, and developmental delay, particularly in consanguineous families.

## Background

Kaufman oculocerebrofacial syndrome (KOS) (OMIM #244450) is a very rare autosomal recessive disorder caused by pathogenic biallelic variants in the *UBE3B* gene. KOS is a multisystem disorder characterized not only by distinctive craniofacial features and global developmental delay, but also by hypotonia, feeding difficulties, growth deficiency, and involvement of multiple organ systems, including the neurologic, ophthalmologic, cardiac, skeletal, and renal systems. Facial features typically include blepharophimosis, ptosis, hypertelorism, short palpebral fissures, low-set ears with preauricular tags, micrognathia, and microcephaly.

Kaufman et al. first described four siblings with a consistent constellation of craniofacial, neurologic, and developmental features in 1971, suggesting an autosomal recessive syndrome. The condition was later named Kaufman oculocerebrofacial syndrome (KOS), and in 2012, biallelic loss-of-function variants in UBE3B were identified as the underlying molecular cause. *UBE3B* encodes an E3 ubiquitin ligase involved in proteasomal degradation and neurodevelopmental regulation [1, 2]. Loss-of-function mutations—including nonsense, frameshift, and splice-site variants—have been implicated in disease pathogenesis, disrupting normal protein function [2, 3].

Since the original molecular discovery, multiple case series and single-case reports from diverse populations have expanded the genotypic and phenotypic spectrum of KOS. However, while no epidemiologic data are available, the diagnosis of KOS may be underrecognized in regions with high consanguinity, such as the Middle East [1, 4]. 

Here, we describe an infant presenting with classical clinical features of Kaufman oculocerebrofacial syndrome (KOS) and a rare homozygous splice site variant in *UBE3B* (c.1741 + 2T > C). Although this variant has been previously reported in ClinVar as pathogenic, no detailed clinical description has been published. To our knowledge, this is the first comprehensive clinical and phenotypic characterization of this variant, further supporting its pathogenicity and emphasizing the importance of molecular diagnostics in syndromic developmental disorders.

## Case presentation

A 12-month-old Jordanian girl was referred for evaluation of global developmental delay and multiple dysmorphic features. She is the fourth child born to healthy consanguineous parents with no known medical conditions. The family history includes a healthy 11-year-old daughter and a 7-year-old son. The second pregnancy, a girl, was delivered prematurely at 33 weeks’ gestation and died at one week of age due to respiratory distress syndrome (RDS) without any further details of her medical condition. There is no history of miscarriages.

Family history is notable for consanguinity, and on the paternal side, one of the father’s sisters had an infant who died at 7 days of age due to an unspecified central nervous system condition. All other extended family members are reportedly healthy, with no known congenital anomalies or neurodevelopmental disorders.

The index patient was delivered via cesarean section at 36 weeks’ gestation. Her birth weight was 2.4 kg (z-score: − 1.0), and her head circumference was 30 cm (z-score: − 1.7), suggesting mild growth restriction. Apgar scores were 5 at 1 min, 6 at 5 min, and 6 at 10 min. She was admitted to the neonatal intensive care unit (NICU) due to dysmorphic features and respiratory distress.

On physical exam, the patient showed multiple dysmorphic features, including microcephaly, hypertelorism, a broad nasal bridge, low-set ears, periauricular skin tags, and micrognathia. Widely spaced nipples were also noted. Additionally, a single café-au-lait macule was observed under the right axilla. Neurological exam revealed generalized hypotonia and overall developmental delay. The anterior fontanelle was open and appropriately sized.

A brain Magnetic Resonance Imaging (MRI), performed as part of the syndromic evaluation during NICU stay, showed no structural abnormalities. Cardiac assessment indicated mild basal septal hypertrophy. Thyroid function tests initially showed low free T4 with normal TSH; repeat testing at 3 months showed normalization of free T4 without intervention. Renal ultrasound was normal. Ophthalmologic exam revealed a normal red reflex and fundoscopic appearance. Audiological screening was normal. Deep tendon reflexes were preserved. No limb abnormalities, such as long digits or joint hyperlaxity, were observed. Serum lipid profile was not assessed.

During infancy, concern arose regarding failure to thrive and delayed developmental milestones. Despite having normal feeding mechanics, she exhibited poor weight gain, likely due to inadequate caloric intake and increased metabolic demands associated with her syndromic condition, prompting the start of a high-caloric formula and feeding. Developmentally, the infant exhibited moderate global delay. Head control was achieved at around 5 months, with rolling at 7 months and sitting unsupported at approximately 11 months. Social smile was noted at 3 months, and babbling emerged at 9 months, but no meaningful words were spoken. Fine motor development was delayed, with limited pincer grasp and minimal object manipulation. She had normal urinary and bowel habits. She continued follow-up in multiple clinics, including genetics, neurology, endocrinology, and cardiology.

Whole-exome sequencing identified a homozygous UBE3B splice-site variant, c.1741 + 2T >C, which we classified as pathogenic according to ACMG/AMP and 2024 ClinGen SVI criteria. The variant disrupts the invariant + 2 donor splice site, fulfilling PVS1 (Very Strong), and is supported by a high SpliceAI score of 0.97 as well as previously demonstrated exon-skipping for similar splice-site variants (Basel-Vanagaite et al., 2012). It is extremely rare in gnomAD v4.1.0 with no reported homozygotes, supporting PM2 (Moderate). In addition, the same variant has been reported in an unrelated affected individual with a compatible phenotype, meeting PS4 (Supporting). ClinVar lists this variant as pathogenic, based on the original Basel-Vanagaite et al. submission [3]. 

Written informed consent was obtained from the patient’s legal guardian for publication of this case report and the accompanying clinical images.

### Genetic testing methods

**Fig. 1:**
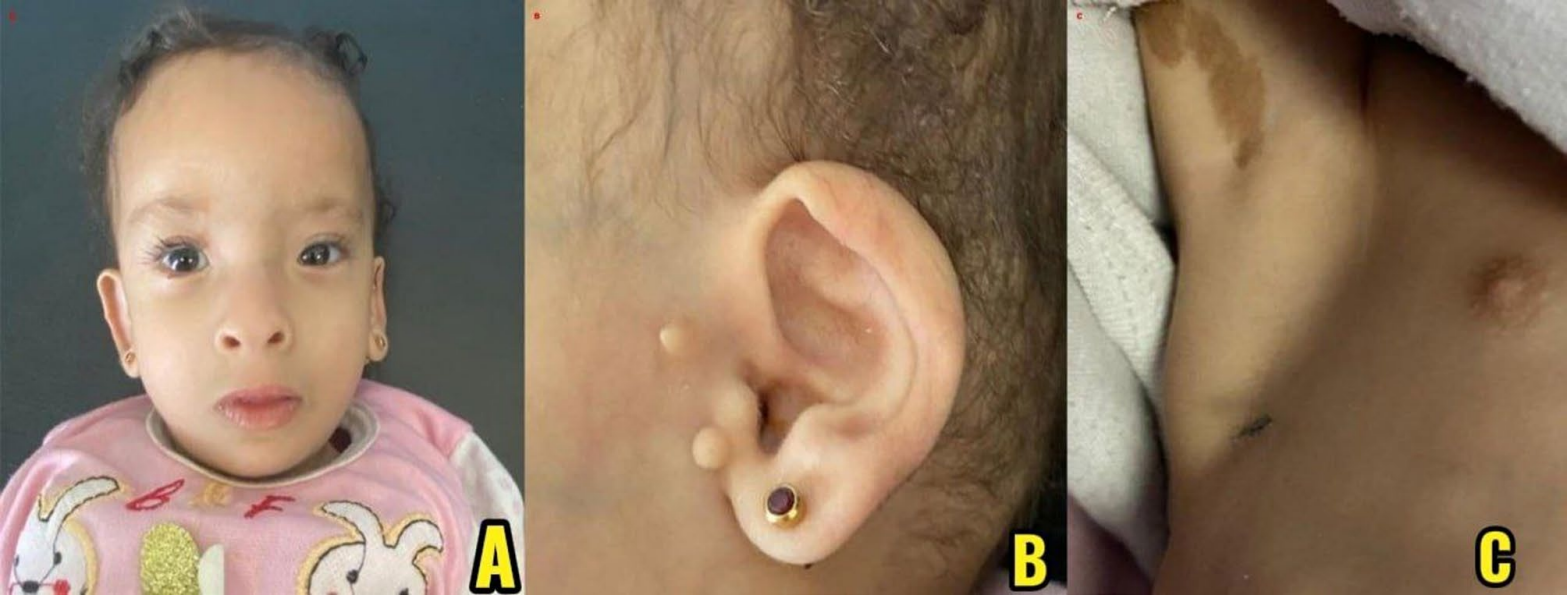
Clinical findings in the patient: (**A**) Frontal view of the patient showing characteristic craniofacial features, including a high forehead, hypertelorism, epicanthal folds, and a short philtrum. (**B**) Lateral view of the right ear showing preauricular skin tags. (**C**) Café-au-lait macule on the right axilla

Genomic DNA was extracted from the patient’s EDTA blood sample and analyzed by whole-exome sequencing (WES). Exome capture was performed using the xGen Exome Research Panel v2, along with the xGen human mtDNA panel and xGen Custom Hyb Panel v1 (Integrated DNA Technologies, USA). Sequencing was conducted on the Illumina NovaSeq X platform, generating approximately 10.4 billion bases, with a mean coverage depth of 152.81× across the targeted 34.2 million bases. Over 99.4% of bases were covered at ≥ 20×, ensuring high-confidence variant detection. Bioinformatics analysis was performed using EVIDENCE v4.2, a proprietary interpretation platform developed by 3 billion Inc. Single-nucleotide variants (SNVs) and insertions/deletions (INDELs) were called using GATK v4.4.0, while Manta v1.6.0 and 3bCNV v2.1 were used for copy number variant (CNV) detection, and ExpansionHunter for repeat expansions. Variants were annotated using Variant Effect Predictor (VEP v104.2) and interpreted according to ACMG/AMP 2015 guidelines, considering both molecular data and patient phenotype. Confirmatory Sanger sequencing was performed for low-confidence variants when necessary (Fig. 1).

## Discussion

KOS is a rare autosomal recessive disorder caused by biallelic pathogenic variants in *UBE3B*, with characteristic craniofacial, developmental, and multisystem features [3, 5]. The syndrome is characterized by features such as distinctive craniofacial dysmorphism, microcephaly, hypotonia, developmental delay, cardiac anomalies, and variable systemic involvement [6]. While many variants in *UBE3B* have been reported, the splice site variant c.1741 + 2T >C is very rare and lacks detailed phenotypic documentation in the literature, despite its classification as pathogenic in ClinVar. Our patient exhibited classic features consistent with KOS, including craniofacial dysmorphism (microcephaly, low-set ears, preauricular tags, and micrognathia), neurodevelopmental abnormalities (global developmental delay, hypotonia), failure to thrive, and mild cardiac anomalies. These features align with prior reports from diverse populations, including those by Yilmaz et al. (2017), Zaki et al. (2020). The identified *UBE3B* variant, c.1741 + 2T >C, is a homozygous splice site mutation disrupting a canonical donor site. With a high SpliceAI score of 0.97 and existing ClinVar pathogenic classification, this variant is strongly predicted to impair splicing and cause loss of function.

While the phenotypic spectrum of KOS is generally consistent, some reports suggest that the nature of the underlying *UBE3B* mutation may influence disease severity. Loss-of-function variants, including nonsense, frameshift, and canonical splice-site mutations such as c.1741 + 2T >C, are typically associated with the classic KOS phenotype characterized by global developmental delay, craniofacial anomalies, and multisystem involvement. In contrast, a few cases with hypomorphic or missense variants have shown variable expressivity or milder features, although this remains an area of ongoing research. Our patient’s presentation aligns with the classical phenotype seen in biallelic loss-of-function cases [2, 3, 7]. 

This splice-site variant has been previously reported as pathogenic, but without detailed phenotypic data. Our case contributes the first comprehensive clinical and phenotypic description of this splice-site variant, confirming its association with classic KOS features. Our patient’s clinical features are generally consistent with previously reported cases of Kaufman oculocerebrofacial syndrome. Similar craniofacial findings, developmental delay, hypotonia, and growth impairment were described in the reports by Yilmaz et al. (2018), Zaki et al. (2020), and Basel-Vanagaite et al. (2012) [2–4]. One notable finding in our case is the presence of mild basal septal hypertrophy, which has not been commonly reported, suggesting that cardiac involvement in KOS may vary between patients. In addition, the family’s consanguinity and the history of neonatal deaths support the possibility of variable expressivity, meaning that individuals carrying the same pathogenic variant may show different degrees of severity or organ involvement. This case therefore adds further clinical detail to the phenotype associated with UBE3B variants. This case highlights the diagnostic utility of whole-exome sequencing in syndromic infants from consanguineous populations and emphasizes the importance of recognizing rare splice-site variants even when phenotypic data is limited.

## Conclusion

We report a 12-month-old infant with Kaufman oculocerebrofacial syndrome (KOS) due to a homozygous UBE3B splice site variant (c.1741 + 2T > C). While this variant has previously been classified as pathogenic, our case provides the first comprehensive phenotypic characterization, thereby adding clinical depth to existing molecular data. The patient’s features were consistent with the classical KOS presentation, and cardiac involvement in the form of mild septal hypertrophy may represent a less frequently described finding. This report highlights the critical role of early genomic testing, particularly whole-exome sequencing, in identifying rare syndromic disorders in consanguineous populations, which can guide clinical management, surveillance, and genetic counseling.

## Data Availability

All data generated or analyzed during this case report are included in this published article.

## Abbreviations

BMRS: Blepharophimosis-Mental Retardation Syndromes
ACMG: American College of Medical Genetics and Genomics
CNS: Central Nervous System
FTT: Failure to Thrive
KOS: Kaufman Oculocerebrofacial Syndrome
MRI: Magnetic Resonance Imaging
NICU: Neonatal Intensive Care Unit
RDS: Respiratory Distress Syndrome
UBE3B: Ubiquitin Protein Ligase E3B
WES: Whole Exome Sequencing
